# Transcriptomic Profiles of Normal Pituitary Cells and Pituitary Neuroendocrine Tumor Cells

**DOI:** 10.3390/cancers15010110

**Published:** 2022-12-24

**Authors:** Jun Y. Oh, Robert C. Osorio, Jangham Jung, Luis Carrete, Nikita Choudhary, Meeki Lad, Atul Saha, Manish K. Aghi

**Affiliations:** Department of Neurosurgery, University of California San Francisco, San Francisco, CA 94143, USA

**Keywords:** pituitary, pituitary neuroendocrine tumors, pituitary adenomas, transcriptomics, scRNA-seq, Cushing, prolactinoma, gonadotroph

## Abstract

**Simple Summary:**

The molecular pathogenesis of pituitary neuroendocrine tumors (PitNETs) involves the interplay of many genes and transcription factors in the setting of a diverse altered tumor microenvironment. Recent advancements in molecular technologies, such as single-cell RNA sequencing, became essential in delineating specific cell types and identifying altered genes in both normal physiological states and diseases in the pituitary. This review discusses the recent progress made in characterizing the transcriptomic profiles of the normal pituitary gland and sporadic PitNETs and their significance.

**Abstract:**

The pituitary gland is one of the most cellularly diverse regions of the brain. Recent advancements in transcriptomic biology, such as single-cell RNA sequencing, bring an unprecedented glimpse into the molecular composition of the pituitary, both in its normal physiological state and in disease. Deciphering the normal pituitary transcriptomic signatures provides a better insight into the ontological origin and development of five types of endocrine cells, a process involving complex cascades of transcription factors that are still being established. In parallel with these observations about normal pituitary development, recent transcriptomic findings on pituitary neuroendocrine tumors (PitNETs) demonstrate both preservations and changes in transcription factor expression patterns compared to those seen during gland development. Furthermore, recent studies also identify differentially expressed genes that drive various tumor behaviors, including hormone hypersecretion and tumor aggression. Understanding the comprehensive multiomic profiles of PitNETs is essential in developing molecular profile-based therapies for PitNETs not curable with current treatment modalities and could eventually help align PitNETs with the breakthroughs being made in applying precision medicine to other tumors.

## 1. Introduction

The pituitary gland is composed of a heterogeneous population of cells that regulate essential homeostatic functions including metabolism, reproduction, and growth. The cell clusters in the anterior lobe of the pituitary gland are mainly characterized by the endocrine hormone that they produce and secrete into blood circulation. Corticotrophs produce adrenocorticotrophic hormone (ACTH), gonadotrophs produce follicular-stimulating hormone (FSH) and luteinizing hormone (LH), somatotrophs produce growth hormone (GH), and lactotrophs produce prolactin (PRL). All pituitary endocrine cells are tightly regulated by upstream hypothalamic neurons and assume different gene expression states in response to internal and external homeostatic cues [1,2].

Pituitary neuroendocrine tumors (PitNETs) (formerly pituitary adenomas) are common intracranial tumors originating from anterior pituitary neuroendocrine cells. PitNETs may cause intracranial mass effects, including headache, dizziness, visual deficits, or deficiency of pituitary hormones [3]. Nearly half of PitNETs produce excess hormones [4]. The signs and symptoms of hypersecreting PitNETs in patients vary depending on their neuroendocrine type. For instance, hypersecreting lactotroph tumor (prolactinoma) causes galactorrhea and suppresses the hypothalamic-pituitary-gonadal axis, resulting in menorrhea, infertility, or erectile dysfunction. Hypersecreting somatotroph tumors cause acromegaly and gigantism. Additionally, hypersecreting corticotroph tumor causes Cushing’s disease, characterized by weight gain, central obesity, skin striae, and facial plethora.

Unlike hypersecreting PitNETs, nonfunctioning PitNETs may be found incidentally or present with signs of mass effect rather than symptoms of excessive hormone secretion. Some nonfunctioning PitNETs are “silent PitNETs” that are immunohistochemistry (IHC)-positive for anterior pituitary hormone and transcription factors, although they do not secrete hormones at a clinically significant level [5]. While most silent PitNETs are silent gonadotrophs [4], all types of hypersecreting PitNETs can have a silent counterpart (i.e., silent lactotroph, silent somatotroph, or silent corticotroph tumors).

The genetic and molecular pathogenesis of PitNETs is diverse. Less than 5% of all PitNETs arise from germline mutations as a part of syndromic diseases (i.e., *MEN1* gene mutation in multiple endocrine neoplasia-1 (*MEN1*)) or as a familial isolated pituitary adenoma) [6]. The remaining 95% of PitNETs develop in the context of sporadic, somatic mutations in various genes that regulate the cell cycle, cell signaling, and transcriptional changes [7], with the lack of ubiquitous change across all PitNETs making it challenging to identify causative genetic changes in these tumors.

Deciphering the molecular characteristics of PitNETs is of particular interest because such information could provide opportunities for unique personalized therapies. Unfortunately, understanding the comprehensive pathogenesis of PitNETs has been difficult mainly due to the interplay of many genes and unique, innate properties of anterior pituitary cell types in the setting of diverse altered tumor microenvironments [8]. Recent advancements in molecular technologies, such as single-cell RNA-sequencing (scRNA-seq) [9,10], became essential in delineating specific cell types and identifying altered genes in both normal physiological states and diseases, especially in a cellularly heterogeneous brain region such as the pituitary. This review discusses the recent progress in characterizing the transcriptomic profiles of the normal pituitary gland and sporadic PitNETs and their molecular implications.

## 2. Normal Pituitary Gland during and after Development

### 2.1. Normal Corticotroph (TPIT-Lineage) Transcription Factors during and after Development

**Figure 1:**
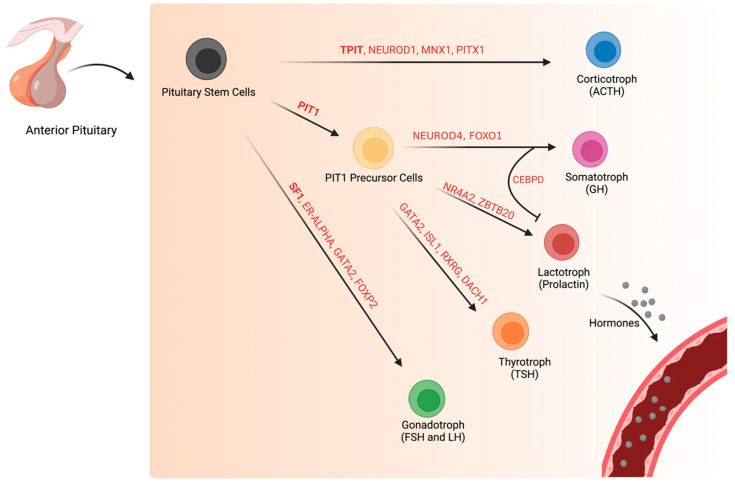
Specific transcription factors drive normal pituitary cell differentiation during development. Pituitary stem cells differentiate into five specialized, endocrine hormone-producing cell types: corticotrophs (ACTH), somatotrophs (GH), lactotrophs (prolactin), thyrotroph (TSH), and gonadotrophs (LH and FSH). Pituitary cell differentiation requires a complex cascade of transcription factors, many of which are still in the process of being identified. Only select transcription factors are highlighted in this diagram.

During normal corticotroph development, *TPIT* (also known as TBX19), a T-box transcription factor, activates the hallmark gene *POMC* with the help of tissue-specific homeodomain transcription factor, *PITX1* [11,12] (Figure 1). Overexpression of *TPIT* in transgenic mice differentiates the pituitary corticotroph lineage, whereas *TPIT* double-knock down results in the loss of corticotroph cells [12,13]. In the absence of *TPIT,* the pituitary neuroendocrine cells that normally differentiate into corticotrophs differentiate instead into gonadotrophs or thyrotroph [13]. This suggests that *TPIT* suppresses altered cell lineage fates.

In the human fetal pituitary, scRNA-seq shows a peaked expression of *NEUROD1*, a basic helix-loop-helix (bHLH) transcription factor gene, at the intermediate states of the corticotroph lineage [14]. *NEUROD1* may be required for the appropriate timing of corticotroph differentiation [15], although it may not be necessary for corticotroph lineage commitment [16]. In mice, *NEUROD1* knock-down results in transient delayed corticotroph differentiation but no changes in *TPIT* expression [16]. Thus, unique genes may regulate specific aspects of normal pituitary cellular differentiation, such as appropriate timing.

Furthermore, scRNA-seq of postmortem pituitaries of pediatric and adults identifies *MNX1* (also known as *HLXB9*) as the second highest significant transcription factor, following *TPIT*, in POMC cells [17]. In the same study, *MNX1* and *TPIT* co-localizes via in situ mRNA hybridization in adult mice pituitary. *MNX1* is involved in developing pancreatic beta cells [18] and motor neuronal cells [19], but its role in corticotroph differentiation remains largely unexplored.

### 2.2. Development of Normal Pituitary Cells of the PIT1-Lineage

*PIT1* (also known as POU1F1), a POU domain-containing transcription factor, is essential for the terminal cell differentiation and gene expression of normal pituitary cells that go on to secrete the neuroendocrine hormones *PRL, GH*, and *TSH* [11]. The formation of these normal pituitary cells of the PIT1 lineage is positively regulated by *PROP1* and negatively regulated by *HESX1* [20].

### 2.3. Normal Somatotroph Transcriptional Pathways during and after Development

In normal somatotrophs, *NEUROD4* (also known as *MATH3*), a PIT1 target gene, is involved in cell maturation and proliferation. *NEUROD4* double-knockout mice show specific downregulation of *GH* and growth hormone-releasing hormone receptor (*GHRHR*) [21]. However, the blockage of somatotroph maturation in the *NEUROD4* knock-out mice may be transient. GH-positive cells, albeit in reduced numbers, are still detectable postnatally in the *NEUROD4* knock-out mice, although the expression of *GHRHR* remains minimal [21]. These findings suggest that *NEUROD4*, similarly to *NEUROD1* in corticotrophs, may regulate the appropriate timing of somatotroph differentiation rather than terminal cellular fate.

Among other genes, scRNA-seq shows upregulation of *FOXO1* and *CEBPD* in fetal human somatotrophs [14]. Pituitary-specific double knock-out of *FOXO1* in mice results in delayed terminal differentiation of somatotrophs and reduced expression of *GHRHR* [22]. Additionally, *FOXO1* knock-out results in decreased expression of *NEUROD4*. Thus, *FOXO1* may be an upstream regulator of *NEUROD4* during somatotroph differentiation [22]. However, this mechanism is still unclear, as scRNA-seq in human fetal somatotrophs suggests that *NEUROD4* is upregulated before *FOXO1* expression [14]. The temporal sequence and dynamics of *NEUROD4* and *FOXO1* and other co-factors involved in this transcriptomic regulatory network are largely unestablished.

Interestingly, the overexpression of *CEBPD* in somatotrophs suggests that differentiating pituitary cells actively repress altered cellular fates. In cell culture, overexpression of *CEBPD* results in the inhibition of PRL expression and lactotroph proliferation, whereas *CEBPD* knockdown using small interfering RNA leads to increased PRL expression [23]. Thus, it is plausible that somatotrophs continuously express *CEBPD* to inhibit other PIT1 lineage differentiations. This is particularly of interest because the traditional model of pituitary cell differentiation generally views lineage-specific transcription factors as promotors of cellular fate, whereas inhibitory mechanism, such as the one seen with *CEBPD*, is not well recognized. Transcription factors may allow cellular differentiation bi-directionally promoting cell fate and repressing altered fate- which may explain why cell fates are so resistant to change.

Recent studies suggest that somatotroph cells are not homogeneous but rather heterogenous in transcriptomic signature. SnRNA-seq of adult mice pituitary shows two distinct sub-clusters of somatotrophs [24]. In the same study, a single nucleus assay for transposase-accessible chromatin with sequencing (snATAC-seq) also shows cluster-dependent polarity in the promotor accessibility regions. In another study, scRNA-seq of adult mice pituitary identifies a subpopulation of somatotrophs that expresses genes involved in sterol and cholesterol-biosynthesis [25]. While the exact significance of heterogeneous gene expression and chromatin accessibility within somatotrophs remains unclear, these molecular signatures may represent distinct functional cell states that allow dynamic neuroendocrine responses to homeostatic stimuli. For instance, growth hormone secretion by the somatotroph cells is a dynamic process involving both pulsatile patterns over a minute timescale and chronic fluctuations associated with growth or aging [26]. Generating distinct cell subpopulations with unique growth hormone release patterns may be one of the possible mechanisms behind secretory plasticity.

### 2.4. Normal Lactotroph Transcriptional Pathways during and after Development

In normal lactotrophs, transcription of the hallmark gene *PRL* requires the Ras-activation of PIT1 [27]. *POU4F1* and *NR4A2* also increase *PRL* expression in PIT1 cells. NR4A2 directly binds to the *PRL* promotor, whereas POU4F1 enhances *PRL* expression without binding to the *PRL* promotor, possibly through unidentified regulatory elements [28]. Co-expression of *POU4F1* and *NR4A2* results in more robust *PRL* expression than seen with *POU4F1* alone, suggesting their synergistic effect on *PRL* [28]. Notably, *POU4F1* seems required for lactotroph differentiation, as *NR4A2* without *POU4F1* is insufficient to increase *PRL.*

In addition, scRNA-seq of the human fetal pituitary identifies upregulation of *ZBTB20* in lactotrophs compared to somatotrophs and thyrotroph [14]. *ZBTB20* knock-down in vitro results in decreased *PRL* promotor activity, whereas *ZBTB20* overexpression, in the presence of PIT1, results in increased *PRL* promotor activity [29]. In *ZBTB20* knock-down mice, lactotroph development is inhibited at the early stage of somatolactotropic precursor cells [29]. These studies suggest that *ZBTB20* regulates lactotroph specification, likely at the somatolactotropic stage, although the exact mechanism is still not well understood. In addition to its vital function in differentiating lactotrophs, *ZBTB20* is critical for PRL expression in mature lactotrophs, at least in mice [30].

Single-cell transcriptomic analysis of adult mice and rat pituitaries points to significant sex-specific differences in gene expressions in lactotrophs. This is not surprising given that sexual maturation, reproduction, and development require sex-dependent endocrine demands and regulation. In addition to expressing a higher level of *PRL,* female lactotrophs, compared to male lactotrophs, express more genes involved in neurotransmission and endocrine release machinery, such as *drd4* (dopamine receptor), *gal* (galanin), and *grik1* (ionotropic glutamate receptor, type subunit 1) [31,32]. While it is difficult to determine whether these sex-specific transcriptomic differences reflect a transient cellular status, such as physiological change during lactation, or permanent status resistant to change, they point to further cellular diversity and complexity that permits pituitary-peripheral homeostasis in a normal physiological state. In another study, the PIT1 cell cluster in adult female mice shows a relative predominance of lactotrophs over somatotrophs [33], suggesting that pituitary cell-type compositions are also different in females and males. Thus, future studies examining pituitary function and disorders, including pituitary neuroendocrine tumors, should carefully consider baseline sex differences in pituitary hormone expression and cell-type composition that may bias interpretations.

### 2.5. Normal Thyrotroph Transcriptional Pathways during and after Development

In normal thyrotrophs, transcription factors *GATA* and *PIT1* interact to drive *TSHβ* gene expression [34,35]. ScRNA-seq on human fetal pituitary shows that the transcription of *GATA2* may involve two SoxC family genes, *SOX4* and *SOX11*, which are expressed before *GATA2* expression and bind to the regulatory region of *GATA2* in thyrotroph precursor cells [14]. Bulk RNA-seq and ATAC-seq in thyrotroph cell cultures show high *GATA2* expression in thyrotroph cells and a large area of accessible chromatin upstream of *GATA2,* respectively [35]. Pituitary-specific *GATA2* knock-out mice show transient loss of thyrotroph cells and decreased production of TSH in response to hypothyroidism induction, in addition to compromised gonadotroph function [36]. However, the eventual recovery of the thyrotroph cell population in mature *GATA2* knock-out mice indicates that *GATA2* alone does not dictate thyrotroph cell fate and maintenance. In the same study, *GATA2* knock-out mice exhibit elevated levels of other transcripts such as *GATA3*, pointing to a possible feedback regulation or compensation mechanism in thyrotroph specification. Given that some pituitary lineage-specific transcription factors seem dispensable rather than required for terminal cell fate, the pituitary transcription cascade may not be linear as traditionally modeled but rather a multi-directional network involving multiple transcription factors with subtle influence and innate compensatory mechanisms.

The transcription factor *ISL1* is expressed in differentiating and postnatal thyrotroph cells [37]. It is involved in thyrotroph function, suggested by increased *ISL1* transcripts in a mice model of thyrotroph hyperplasia (*Cga*-/-) [37]. However, while deleting *ISL1* in TSH-Cre mice results in a phenotype resembling hypothyroidism, it does not entirely ablate thyrotroph cells, similar to *GATA2* knock-out mice [37]. One explanation could be that transcription factors such as *ISL1* may be more critical for cell functionality than cell differentiation, although delineating these differences may be difficult. Additionally, as previously mentioned, multiple transcription factors may simultaneously maintain normal homeostasis, and experimentally manipulating (i.e., knock-out) one transcription factor at a time, while sufficient to decrease cellular function such as hormone secretion, may not be enough to ablate a pituitary lineage.

ScRNA-seq on the human fetal pituitary identifies *RXRG* and *DACH1* as two enriched genes in thyrotroph cells [14]. They are both upregulated early in precursor PIT1 cells and continue to be upregulated in mature thyrotroph cells. In contrast, *RXRG* and *DACH1* are downregulated in the other two terminal PIT1 cell lines (i.e., somatotrophs and lactotrophs). Thus, *RXRG* and *DACH1* may be specific for thyrotroph development and serve as potential transcriptomic markers to delineate thyrotroph from somatotroph and lactotroph. Whether targeted silencing of *RXRG* and *DACH1* in differentiating thyrotroph cells is sufficient to reduce or revert its neuroendocrine cellular phenotype remains unanswered.

### 2.6. Normal Gonadotroph (SF1-Lineage) Transcriptional Factors during and after Development

Differentiation of normal gonadotrophs involves *SF1* (also known as *NR5A1*), a zinc finger nuclear receptor that regulates multiple sex determination and reproduction genes in adrenal glands, gonads, and hypothalamus [11,38]. The transcription of *SF1* partially depends on the binding of estrogen to estrogen-receptor-alpha (ER-alpha), which mediates the chromatin remodeling of the *SF1* enhancer and promotor in vitro [39]. ScRNA-seq and chromatin accessibility assays on human pituitary cells show ER-alpha as the most significant transcription factor associated with *SF1* expression [17]. Pituitary-specific *SF1* knock-out in *alpha*-*GSU*-Cre mice results in hypogonadism and decreased expression of LH and FSH. Interestingly, *SF1* may be important but not necessary for gonadotroph differentiation since a high dose of exogenous GnRH can rescue the loss of *LH* expression in *SF1* knock-out mice [38].

*GATA2*, expressed earlier than *SF1*, also plays a role in gonadotroph differentiation since *GATA2* inactivation results in reduced gonadotrophin expression [36]. In addition, scRNA-seq of mice pituitary shows enrichment of transcripts encoding *FOXP2*, a novel forkhead homeobox transcription factor, in the gonadotrophs [25]. *FOXP2* co-localizes with LH and FSH in vivo [25], but its exact role in gonadotroph function and differentiation remains to be elucidated.

In the human fetal pituitary, pseudotime analysis, which infers the ordering of cells in a lineage based on gene expression profiles of scRNA-seq, reveals two multi-step developmental trajectories that contain a *GATA*-positive/*NR5A1*-negative, “pre-gonadotroph” intermediate cell state [14]. The intermediate cell differentiates into two subtypes of *NR5A1*-positive gonadotrophs characterized by different expression patterns of chorionic gonadotropin and FSH hormones. One of the subtypes is enriched with differentially expressed genes involved in the hormone biosynthesis process and exocytosis [14]. This suggests that there are heterogenous gonadotroph populations with specific functions, such as secretion of specific hormones, at least during the development stage, that may play a role in establishing the early hypothalamic-pituitary-gonadal axis [14]. Intriguingly, in the same study, a subset of gonadotrophs also expresses *MC2R*, an ACTH receptor, suggesting that there may be crosstalk between gonadotroph and corticotroph lineage cells during development. Likewise, a subset of intermediate corticotrophs expresses some gonadotroph-related markers [14]. Whether gonadotroph-corticotroph crosstalk continues to exist in healthy adult human pituitary glands remains unclear.

## 3. The Transcriptomic Landscape of PitNETs

The pathogenesis of PitNETs is a complex process that involves abnormal transcriptomic changes, among other intrinsic and extrinsic drivers, that result in cell cycle dysregulation, loss of tumor suppressor factors, and signaling defects [7,40]. PitNETs are classified by their lineage-restrictive pituitary transcription factors (i.e., TPIT for corticotroph adenomas; PIT1 for somatotroph, lactotroph, and thyrotroph adenomas; SF1 for gonadotroph adenomas; and absence of PIT1, TPIT, and SF1 for null cell adenomas), which were first delineated in the 2017 World Health Organization (WHO) classification guideline and preserved in the 2022 guidelines [41,42]. Recent studies examining state-dependent cellular transcriptomic changes provide a deeper insight into the complexity of PitNET tumorigenesis and help define which of these changes reflect the hijacking of developmental transcriptomic programming and which are novel to PitNET tumorigenesis.

### 3.1. Corticotroph PitNETs

In 30–60% of ACTH-secreting PitNETs, dysregulation of ubiquitin-specific protease 8 (USP8) and the epidermal growth factor receptor (EGFR) pathway, neither of which are implicated in fetal corticotroph development, play a significant role [43,43]. Whole-exome sequencing of human corticotroph PitNETs reveals somatic mutations in *USP8* [44,45,46]. *USP8* mutations are specific to corticotroph PitNETs; other PitNETs do not display USP8 mutations on targeted sequencing [44]. In normal conditions, USP8 deubiquitinates various proteins, including EGFR, and prevents their lysosomal degradation [47,48]. *USP8* gain-of-function mutations result in the elevated deubiquitinating activity of EGFR and thereby increased accumulation of EGFR in the plasma membrane [44,45]. Enhanced EGFR signaling increases *POMC* transcription and ACTH secretion in the mice corticotroph adenoma cell line (i.e., AtT20) [49]. In contrast, EGFR inhibitor (i.e., gefitinib) suppresses *POMC* transcription in cultured cells derived from human corticotroph PitNETs [49]. Consistent with these findings, *USP8-*mutated corticotroph PitNETs show a higher incidence of *EGFR* expression, and *USP8* knock-down reduces in vitro ACTH secretion [44].

In contrast, whole-exome sequencing studies in corticotroph PitNETs without USP8 mutations (i.e., USP8-wildtypes) identify BRAF and USP48 mutations. Similarly to USP8, BRAF and USP48 mutations are unique to corticotroph PitNETs. BRAF and USP48 mutations increase in vitro *POMC* transcription and potentiate the stimulation effect of upstream cortisol-releasing hormone (CRH) [50,51]. In addition, patients with *BRAF* mutations have higher plasma ACTH and cortisol [50]. While these findings are encouraging for developing targeted therapies, whether targeting USP8, USP48, and BRAF is sufficient to attenuate Cushing’s disease phenotype in vivo remains unanswered.

As previously mentioned, transcription factor *NEUROD1* peaks at the intermediate states of corticotroph lineage development in the human fetal pituitary, whereas *MNX1* remains upregulated in corticotroph cells in normal human adult pituitary. Bulk-RNA seq of corticotroph PitNETs shows that *MNX1* is mildly upregulated, whereas *NEUROD1* expression remains low [52], suggesting that corticotroph PitNETs may represent a well-differentiated cellular state. In contrast, a scRNA-seq study of corticotroph PitNETs indicates the opposite, possibly pointing to a de-differentiated cellular state, with upregulation of *NEUROD1* [53]. These differences in the studies may be due to different methodologies employed (i.e., bulk-RNA seq capturing multiple cell types in addition to corticotroph tumor cells) or intertumoral variations from patients. More studies comparing the temporal sequences of lineage-specific transcription factor expressions in PitNETs, using tools such as lineage tracing [54], to those of healthy cells will be essential to clarify these differences.

**Figure 2:**
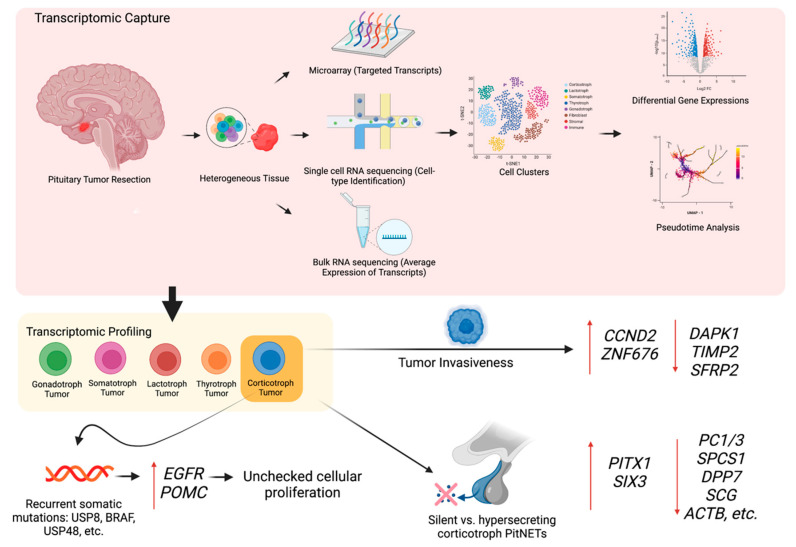
Transcriptomic profiling of pituitary neuroendocrine tumors. (TOP) A transcriptome captures a snapshot in time of diverse transcripts present in a pituitary tumor cell. Microarrays capture a set of predetermined sequences whereas single-cell RNA sequencing and bulk RNA sequencing capture all transcripts using high-throughput sequencing. In contrast to bulk RNA sequencing, single-cell RNA sequencing can identify and analyze cell subpopulations. (BOTTOM) Transcriptomic analysis reveals differentially expressed genes that drive various tumor behaviors, such as hormone hypersecretion and tumor invasiveness. Transcriptomic changes in a corticotroph tumor cell are highlighted in this diagram.

Corticotroph PitNETs exhibit heterogenous phenotypes, including variability in tumor aggressiveness and the ability to secrete ACTH. Transcriptomic analysis of corticotroph PitNETs suggests that tumor behavior correlates with differentially expressed genes (Figure 2). Compared to non-invasive corticotroph PitNETs, invasive corticotroph PitNETs exhibit upregulation of cyclin D2 (*CCND2*) and zinc-finger protein 676 (*ZNF676*) and downregulation of death-associated protein kinase 1 (*DAPK1*) and TIMP metalloproteinase inhibitor 2 (*TIMP2*) in microarray analysis and qRT-PCR validation [55]. *CCND2* and *DAPK1* regulate the cell cycle and *TIMP2* regulates extracellular matrix homeostasis [56,57]. *ZNF676* plays a role in telomere homeostasis but the involvement of telomere dysregulation in PitNETs pathogenesis remains debated [58,59]. In another study, bulk RNA-seq reveals the downregulation of secreted frizzled-related protein 2 (*SFRP2*) in invasive corticotroph PitNETs [60]. *SFRP2* regulates Wnt signaling pathway and acts as a tumor suppressor [60,61].

Comparing silent and hypersecreting corticotroph PitNETs using scRNA-seq exhibits robust transcriptomic differences in their tumor cells. Compared to the hypersecreting corticotroph PitNETs, silent corticotroph PitNETs show significantly lower levels of genes involved in prohormonal processing (i.e., prohormone convertase (*PC1/3*), signal peptidase (*SPCS1*), and dipeptidyl peptidase (*DPP7*)), secretory vesicle regulation (i.e., granin proteins (*SCG*)), and exocytosis regulation (i.e., cytoskeleton components (*ACTB*, *PFN1*, *GSN*, *MYL12A*)) [62]. Silent corticotrophs PitNETs exhibit higher expression of organogenesis genes (i.e., *PITX1*, *SIX3*) which suggests a de-differentiated cellular state. In addition, silent corticotroph PitNETs exhibit characteristics of epithelial to mesenchymal transition (i.e., N-cadherin (*CDH2*) and mesenchymal matrix markers (*COL1A1*, *COL4A1*)), pointing to an increased tumor migration potential [62,63]. Consistent with these findings, the stromal cells in the silent corticotroph PitNETs, but not in hypersecreting corticotroph PitNETs, exhibit high markers of vascular smooth muscle cells and pericytes, reflecting a microenvironment that promotes angiogenesis [62]. Thus, further mechanistic studies examining the unique, bidirectional interplay between pituitary tumor cells and the microenvironment, including stromal dysregulation, may be essential in understanding the tumorigenic processes.

A recent transcriptomic study suggests proteasome-apoptosis pathway plays a role in corticotroph PitNET pathogenesis. ScRNA-seq of hypersecreting corticotroph PitNETs reveals upregulation of *PMAIP1*, which encodes a pro-apoptotic noxa protein [64]. Interestingly, despite both transcriptional and epigenetic upregulation of *PMAIP1,* the hypersecreting corticotroph PitNETs evade apoptosis through proteasomal degradation of noxa protein. Consistently, proteasomal inhibitors (i.e., bortezomib) rescue the noxa protein and inhibit the growth of patient-derived corticotroph tumor cell lines [64]. While in vivo studies are warranted, the proteasome system in Cushing’s disease may be a potential drug target.

### 3.2. Somatotroph PitNETs

Previous studies have identified somatic mutations in *GNAS*, a gene that is part of signaling in normal fully developed somatotrophs but not in developing fetal somatotrophs, in up to 40% of somatotroph PitNETs. These mutations have been gain-of-function *GNAS* mutations resulting in constitutive activation of adenylyl cyclase and autonomous secretion of growth hormone [65,66,67,68]. *GNAS* mutations are more commonly found in smaller-sized somatotroph PitNETs and are associated with the densely granulated variant on histology [69]. Some studies suggest that *GNAS* mutation is associated with a better response to somatostatin receptor analogs but these findings are not consistent in the literature and require further investigation [46,70,71,72]. In a bulk-RNA seq study, *GNAS*-mutated somatotroph PitNETs are associated with higher expression of *D2R*, suggesting that dopamine agonists may benefit a subtype of acromegaly patients [52]. Another bulk-RNA seq study shows that specifically somatostatin receptor 5 (*SSTR5*) is overexpressed in somatotroph PitNETs [73], partially explaining the superior efficacy of pasireotide which has a higher affinity for SSTR5 over SSTR2 [74]. Somatostatin and dopamine receptors have also been proposed to interact and form distinct complexes (i.e., heterodimerization [75]) that inhibit adenylyl cyclase, limiting growth hormone secretion and proliferation [76,77,78]. However, it remains unclear if this mechanism is of biological or therapeutic significance in PitNETs. Further characterization of somatostatin and dopamine receptor expression profiles, including their receptor subtypes and molecular interactions, in medication-susceptible and medication-resistant somatotroph PitNETs, may provide an opportunity to design the optimal receptor-targeting therapeutic strategy for these tumors.

Similar to normal developing somatotroph cells, *NEUROD4* is upregulated in somatotroph PitNETs [52,53]. As previously mentioned, *NEUROD4* is a key transcription factor that works closely with *FOXO1* in the terminal differentiation of normal somatotrophs, although whether *NEUROD4* is upstream or downstream of *FOXO1* is debated. Interestingly, despite *NEUROD4* upregulation, there seem to be no significant changes in *FOXO1* expression in somatotroph PitNETs [52]. This suggests that somatotroph PitNETs may employ a FOXO1-independent pathway for cell proliferation, perhaps recruiting other downstream transcription factors yet to be identified. It is also possible that in the setting of unchecked cell proliferation, FOXO1 may be suppressed by a negative feedback mechanism.

A recent scRNA-seq study identifies high expressions of *GHRHR*, *GH1*, and *GH2* in somatotroph PitNETs, consistent with their hyperfunctioning status [53]. Furthermore, compared to normal somatotroph cells, additional genes involved in the hormone exocytosis (i.e., *CG3*, *ANXA2*, *CLU*, and *GAA*) and secretion (i.e., *A1BG*, *HEXB*, *ATP6V0A1*, *ATP6AP1*, *PSAP*, and *PSMA5)* are upregulated. The same study identifies upregulation of *AMIGO2* in somatotroph PitNETs, as well as in gonadotroph and lactotroph PitNETs. AMIGO2, a transmembrane protein, has been previously implicated in the pathogenesis of other cancers such as melanoma [79] but its mechanism in PitNET pathogenesis needs further investigation. Given that majority (~76%) of differentially expressed genes are upregulated in somatotroph tumor cells, it would be of interest to examine abnormal changes in chromatin accessibility that are affecting global gene expression.

### 3.3. Lactotroph PitNETs

In 20% of sporadic lactotroph PitNETs, a somatic mutation in splicing factor 3 subunit B1 (*SF3B1*) is a defining genetic signature [80]. The *SF3B1* mutation does not appear in other types of PitNETs and is not implicated in fetal lactotroph development, although it is involved in diverse non-pituitary tumors [81,82,83]. RNA-seq in lactotroph PitNETs with the *SF3B1* mutation shows increased estrogen-related receptor gamma (ESRRG) expression [80]. This gain-of-function mechanism likely involves aberrant alternative splicing [84,85]. Notably, the ESRRG in the *SF3B1* mutant group has a higher affinity for PIT-1 and more robust transcriptional activation of *PRL* than canonical ESRRG, resulting in enhanced cell proliferation [80]. *SF3B1* mutation also downregulates Discs large 1 (*DLG1*), a tumor suppressor gene, in lactotroph PitNETs, thereby promoting tumor cell migration and invasion in vitro [85].

As mentioned before, *ZBTB20* plays a vital role in normal lactotroph cells during development and maturity. More specifically, it is involved in *PRL* expression. Given that most lactotroph PitNETs are functional and secrete prolactin, it may seem likely that *ZBTB20* is highly expressed in lactotroph PitNETs. However, *ZBTB20* does not seem to be differentially expressed in lactotroph PitNETs, as compared to other PitNET types [52], although direct comparisons with normal lactotroph cells are not available. Unlike normal lactotroph cells, prolactin hypersecretion in lactotroph PitNETs may be *ZBTB20*-independent and likely depend on other transcription factors.

Classical animal model studies suggest that dopamine signaling may play a significant role in the pathogenesis of lactotroph PitNETs. Mice lacking dopamine 2 receptors (D2Rs) show hyperprolactinemia and the development of lactotroph adenoma [86,87], whereas mice lacking dopamine transporter (DT), which increases dopaminergic tone, exhibit reduced numbers of lactotrophs and somatotrophs [88]. To date, these murine studies have not been corroborated by any studies identifying *DRD2* gene mutations in human lactotroph PitNETs [89,90].

In the subpopulation of lactotroph PitNETs resistant to dopamine agonist medications (i.e., cabergoline and bromocriptine), studies suggest that there is a decreased expression of D2R isoform [91,92] and inhibitory G protein subunit (G_αi_), a downstream of D2R [93]. However, there seem to be no changes in the affinity of the dopamine agonist for the D2Rs [94]. Furthermore, RT-PCR of a few targeted genes shows that nerve growth factor-B receptor (NGFR) is also expressed less in drug-resistant lactotroph PitNETs [95]. NGFR regulates the expression of D2Rs via the NF-kB intracellular signaling pathway [96,97]. Low NGFR may suppress D2R expression, predisposing an individual to a dopamine agonist-resistant state. Nevertheless, the molecular underpinning of drug-resistant vs. drug-susceptible lactotroph PitNETs remains largely unknown. It likely involves additional genes that modulate the functional expression of D2Rs (i.e., receptor presentation, internalization, degradation, trafficking) and non-D2R-related factors (i.e., increased estrogen or prolactin-receptor).

Microarray transcriptomic studies have identified genes that are differentially expressed in non-invasive vs. aggressive-invasive lactotroph PitNETs. Non-invasive lactotroph PitNETs exhibit downregulation of genes implicated in proliferation (i.e., CENPE, PTTG, and CCNB1) and upregulation of the gene (i.e., KIF13B) associated with tumor-suppressor DGL1 [98,99]. Notably, PTTG is an oncogene involved in various cancers and associated with metastatic potential [100,101,102,103]. In contrast, aggressive-invasive lactotroph PitNETs exhibit downregulation of PITX1, a pituitary development transcription factor, and SCN3B, a sodium channel subunit involved in p53- dependent apoptotic pathway [98]. In addition, they exhibit upregulation of metalloproteinase (i.e., ADAMTS6). The role of ADAMTS6 in tumor development is unclear, as it seems to act as either a tumor suppressor or pro-tumoral agent, depending on the cancer type [104]. While molecularly defining the invasiveness of prolactinoma remains challenging, future multiomics studies at a single-cell level are encouraged to capture further subtle but substantial transcriptomic variations that drive the heterogeneous lactotroph tumor behavior in well-characterized patients.

### 3.4. Thyrotrophic PitNETs

Given their rarity, representing ~1% of all PitNETs, the molecular mechanism of thyrotroph PitNET pathogenesis has not been well defined to date. Unlike corticotroph and somatotroph PitNETs, recurrent somatic mutations have yet to be identified in thyrotroph PitNETs [105]. In studies with limited samples, thyrotroph PitNETs do not exhibit activating mutations of genes encoding G protein subunits, including alpha q and alpha 11, or thyrotropin-releasing hormone receptors [106]. However, altered gene expression involving downstream components that drive tumorigenesis cannot be ruled out. Some studies point to somatic mutations of thyroid receptor-beta resulting in impaired thyroid-mediated negative regulation [107]. In contrast, others suggest aberrant gene expression of thyroid receptor-beta without somatic mutations [108]; thus, these findings are debated. In a bulk RNA-seq study, thyrotroph PitNETs share transcriptomic signatures with both plurihormonal PIT-1 positive adenomas and sparsely granulated somatotroph PitNET [52], suggesting a subtle discrepancy between the WHO histology classification and transcriptome profiling, with the transcriptomic profiling suggesting greater overlap between subtypes than the immunostaining used to define WHO subtypes.

### 3.5. Gonadotroph PitNETs and Null Cell PitNETs

Gonadotroph PitNETs (which are usually clinically silent and do not hypersecrete the FSH or LH hormones associated with their lineage) and null cell PitNETs are grouped together here because there remains some uncertainty about their classification and some overlap between them. While 2017 WHO classification of pituitary tumors differentiating silent gonadotroph tumors (SF1-positive) from null cell tumors (negative for cell type-specific transcription factors) is of clinical relevance given more invasiveness and worse clinical outcome in null cell adenomas [5,109,110], studies suggest that the transcriptomic profiles of null-cell adenomas and gonadotroph PitNETs may not be too different. Unsupervised classification of PitNETs by bulk RNA-seq shows the inclusion of null-cell adenomas (*n* = 8) in the gonadotroph PitNET (*n* = 29) cluster [52]. ScRNA-seq of PitNETs with one null cell adenoma generally supports this finding [53]. Thus, further discussion on whether the current category of gonadotroph PitNETs should also include null-cell adenomas may be warranted. Furthermore, gonadotroph transcriptomic signatures are found in a subset of silent corticotroph and somatotroph PitNETs, suggesting the possibility that gonadotroph PitNETs themselves may not warrant a separate subtype [52].

As previously mentioned, there is evidence for crosstalk between gonadotroph and corticotroph lineage cells during normal fetal pituitary development [14]. If such crosstalk persists during adulthood, the cell types with both gonadotroph and corticotroph characteristics may possibly be the cells of origin for gonadotroph-corticotroph tumor cells. Alternatively, if the gonadotroph-corticotroph crosstalk is a transient phenomenon only during fetal development- representing an intermediate cellular state-, gonadotroph-corticotroph PitNETs may reflect a more de-differentiated state.

The nonfunctional status of silent gonadotroph PitNETs is reflected by the downregulation of key genes involved in hormone production. For instance, compared to normal adult pituitary cells identified from PitNET resection, scRNA-seq of silent gonadotroph tumor cells exhibit downregulation of *LHB*, *ESR1*, and *GNRHR* [53]. This contrasts with somatotroph PitNETs, which are often functional, showing high hormone gene expression [53]. In the same study, most genes are downregulated in gonadotroph PitNETs, including genes involved in regulating cell proliferation (i.e., *CDKN1A*, *CDKN2A*, *ZFP36*, *BTG2*, *DLG5*, and *ZBTB16*) and epithelial development (i.e., *KRT8*, *KRT18*, and *KLF4*). Thus, gonadotroph PitNETs may not just be hormonally silent but may represent a subtype of PitNETs with more globally diminished gene expression, which could explain why they overlap so much with null cell adenomas. Examining epigenetic regulation, especially silencing by DNA methylation, may provide more details on the mechanism of this diminished gene expression.

Transcriptomic profiling also identifies differential gene expression patterns in aggressive- and non-aggressive silent gonadotroph PitNETs. Microarray analysis of non-functional pituitary adenomas (comprising mostly gonadotroph PitNETs) shows higher expression of *MYO5A* and *IGFBP5* in invasive forms compared to non-invasive forms [111]. *MYO5A* and *IGFBP5* are involved in tumor cell migration and metastasis in other cancers [112,113,114]. In another study, bulk RNA-seq of gonadotroph PitNETs shows that genes regulating epithelial-mesenchymal transition (i.e., *SPAG9, SKIL, MTDH, HOOK1, CNOT6L*, and *PRKACB*) are particularly highly expressed in fast-growing tumors, as measured by tumor volume doubling time. While requiring further validations, these studies highlight the utility of molecular profiling in identifying potential predictive biomarkers for tumor growth potential.

## 4. Conclusions

Recent advancements in transcriptomic biology, including the development of single-cell level RNA sequencing, have opened opportunities to molecularly dissect the pituitary, one of the most cellularly diverse regions of the brain. Deciphering the normal pituitary transcriptomic signatures gives us a better insight into the ontological origin and development of five types of endocrine cells, a process involving complex cascades of transcription factors that are still in the process of being established. In parallel with these observations about normal pituitary development, recent findings on PitNETs demonstrate both preservations and changes in transcription factor expression patterns. These studies of PitNETs also point to differentially expressed genes that drive various tumor behaviors, including hormone hypersecretion and tumor aggression. Understanding the comprehensive multiomic profiles of PitNETs will be essential in developing molecular profile-based therapies for PitNETs not curable with current treatment modalities and could eventually help align PitNETs with the breakthroughs being made in applying precision medicine to other tumors.
